# Carmustine Wafers Implantation in Patients With Newly Diagnosed High Grade Glioma: Is It Still an Option?

**DOI:** 10.3389/fneur.2022.884158

**Published:** 2022-06-23

**Authors:** Luca Ricciardi, Ivana Manini, Daniela Cesselli, Sokol Trungu, Amedeo Piazza, Antonella Mangraviti, Massimo Miscusi, Antonino Raco, Tamara Ius

**Affiliations:** ^1^UOC di Neurochirurgia, Department of NESMOS, Sapienza University of Rome, Rome, Italy; ^2^Institute of Pathology, University Hospital of Udine, Udine, Italy; ^3^Department of Pathology, University Hospital of Udine, Udine, Italy; ^4^UO di Neurochirurgia, Azienda Ospedaliera Cardinal G. Panico, Tricase, Italy; ^5^Neurosurgery Unit, Department of Neurosciences, S. Maria della Misericordia University Hospital, Udine, Italy

**Keywords:** glioma surgery, Carmustine, extent of resection, overall survival, complications

## Abstract

**Background:**

The implantation protocol for Carmustine Wafers (CWs) in high grade glioma (HGG) was developed to offer a bridge between surgical resection and adjuvant treatments, such as radio- and chemotherapy. In the last years, however, a widespread use of CWs has been limited due to uncertainties regarding efficacy, in addition to increased risk of infection and elevated costs of treatment.

**Objective:**

The aims of our study were to investigate the epidemiology of patients that underwent surgery for HGG with CW implantation, in addition to the assessment of related complications, long-term overall survival (*OS*), and associated prognostic factors.

**Methods:**

Three different medical databases were screened for conducting a systematic review of the literature, according to the PRISMA statement guidelines, evaluating the role of BCNU wafer implantation in patients with newly diagnosed HGG. The search query was based on a combination of medical subject headings (MeSH): “high grade glioma” [MeSH] AND “Carmustine” [MeSH] and free text terms: “surgery” OR “BCNU wafer” OR “Gliadel” OR “systemic treatment options” OR “overall survival.”

**Results:**

The analysis of the meta-data demonstrated that there was a significant advantage in using CWs in newly diagnosed GBM in terms of *OS*, and a very low heterogeneity among the included studies [mean difference 2.64 (95% *CI* 0.85, 4.44); *p* = 0.004; I2149 = 0%]. Conversely, no significant difference between the two treatment groups in terms of PFS wad detected (*p* = 0.55). The analysis of complications showed a relatively higher rate in Carmustine implanted patients, although this difference was not significant (*p* = 0.53).

**Conclusions:**

This meta-analysis seems to suggest that CWs implantation plays a significant role in improving the *OS*, when used in patients with newly diagnosed HGG. To minimize the risk of side effects, however, a carful patient selection based mainly on patient age and tumor volume should be desirable.

## Introduction

Therapeutic, surgical, and genetic refinements have evolved in these past decades, however, High Grade Glioma (HGG) still remains to be the highest-grade malignant primary tumor of the central nervous system with an extremely poor prognosis, especially in patients with grade WHO IV (1–3).

Despite extensive resection, HGG remains almost incurable because of its deep tumoral infiltration, which tends to promote HGG recurrence that generally occurs in the proximity of the original tumor site (4, 5). By virtue of the growing pattern, tumoral HGG cells can be found beyond the infiltrative tumor area intraoperatively detected by 5-ALA fluorescence, thus supporting the role of supramaximal resection, when functionally possible (6–8).

Carmustine wafers (CWs) marketed as Gliadel®, biodegradable copolymers discs impregnated with the alkylating agent (Bis-ChloroethylNitrosoUrea: BCNU), have been developed as a therapeutic bridge during the period between tumoral surgical resection and standard chemo-radiotherapy onset (Stupp regimen) (9–14). The use of CWs, however, represents a controversial topic among neurosurgeons mainly due to the lack of phase III studies in this field (5, 10, 15, 16). In addition, CWs use has been greatly limited for several reasons, including elevated costs, and the precluded enrolment of patients in subsequent clinical trials because the use of CW could give rise to confounding results (5, 11, 15–20).

Although this treatment option seems to have lost clinical importance in the recent few years, current long-term follow-up investigations have demonstrated a survival benefit in newly HGG treated with CWs implantation, shedding thus the light on the effectiveness of this option (21, 22).

The aim of this meta-analysis, which reports the intraoperative implantation of CWs in newly HGG patients, is to investigate its impact in terms of overall survival (*OS*) and progression-free survival (*PFS*) in comparison with standard surgical treatment without CWs. Side effect and complication data were also evaluated and discussed.

## Materials and Methods

### Study Design

The present study is a systematic review of the literature, consistently conducted according to the preferred reporting items for systematic reviews and meta-analyses (PRISMA) statement guidelines.

### Review Question

The review questions, according to the PRISMA statement, were formulated following the PICO (P: patients; I: intervention; C: comparison; O: outcomes) scheme, as it follows:

In newly diagnosed HGG (P), has the intraoperative implantation of CWs (I) revealed as effective when compared to the standard treatment (Stupp Regimen) (C), in terms of *OS* and *PFS* (O)?

### Inclusion and Exclusion Criteria

The investigations were selected according to the following criteria: 96 English language, comparative study on CWs implantation in newly HGG patients, and adult study populations. Exclusion criteria included language other than English, non-comparative studies, and non-reported quantitative data for analysis.

### Search Strategy

Four different medical databases (PubMed, Scopus, Cochrane Library, and Mendeley) were screened for conducting a systematic review of the literature, according to the PRISMA statement, evaluating the role CWs implantation in patients with newly diagnosed HGG.

The search query was based on a combination of medical subject headings (MeSH): “high grade glioma” [MeSH] AND “Carmustine wafer” [MeSH] and free text terms: “surgery” OR “Gliadel” OR “Gliadel” OR “glioblastoma” OR “systemic treatment options” OR “overall survival” OR “side effects.”

Papers reporting incomplete or non-poolable data, such as means missing standard deviations or medians missing interquartile ranges, were excluded or included only for the follow-up periods during which the data were complete. The “Title” and “Abstract” of the papers were independently screened by two authors (A.P. and A.M.).

Duplicated papers were excluded from the screening. In the second review round, papers included for the Full text analysis were screened, and considered for inclusion according to the inclusion criteria. The references of papers considered were then screened for papers erroneously missed in the first round of review round (forward search). Papers not considered as eligible were excluded with reason. Any discordance in the screening process was solved by consensus with a third senior author (T.I.). Included papers were considered for data analysis and evidence synthesis.

### Outcome Measurements

Title, list of authors, year and journal of publication were collected for every included paper. The following outcomes were extracted from the included papers:

- Overall survival: The OS time was defined as extending from surgery until patient death.
- Progression-free survival: The PFS time was defined as extending from surgery until the demonstration of gadolinium enhancement on follow-up imaging.
- Complications.

### Statistical Analysis

Data of the study populations were summarized using proportion and weighed means. The means and standard deviations in individual studies were estimated from the median and interquartile ranges, when needed, according to the method described by Wan et al. (23). Pooled mean differences (PMD) for continuous variables were computed between outcome groups with a random effects model (24). Comprehensive meta-analysis software (Review Manager – RevMan 5.4.1 The Cochrane Collaboration, 2020) was used for pooling data. The *p*-value was considered significant at α < 0.05.

## Results

### Included Studies and Patients

**Figure 1:**
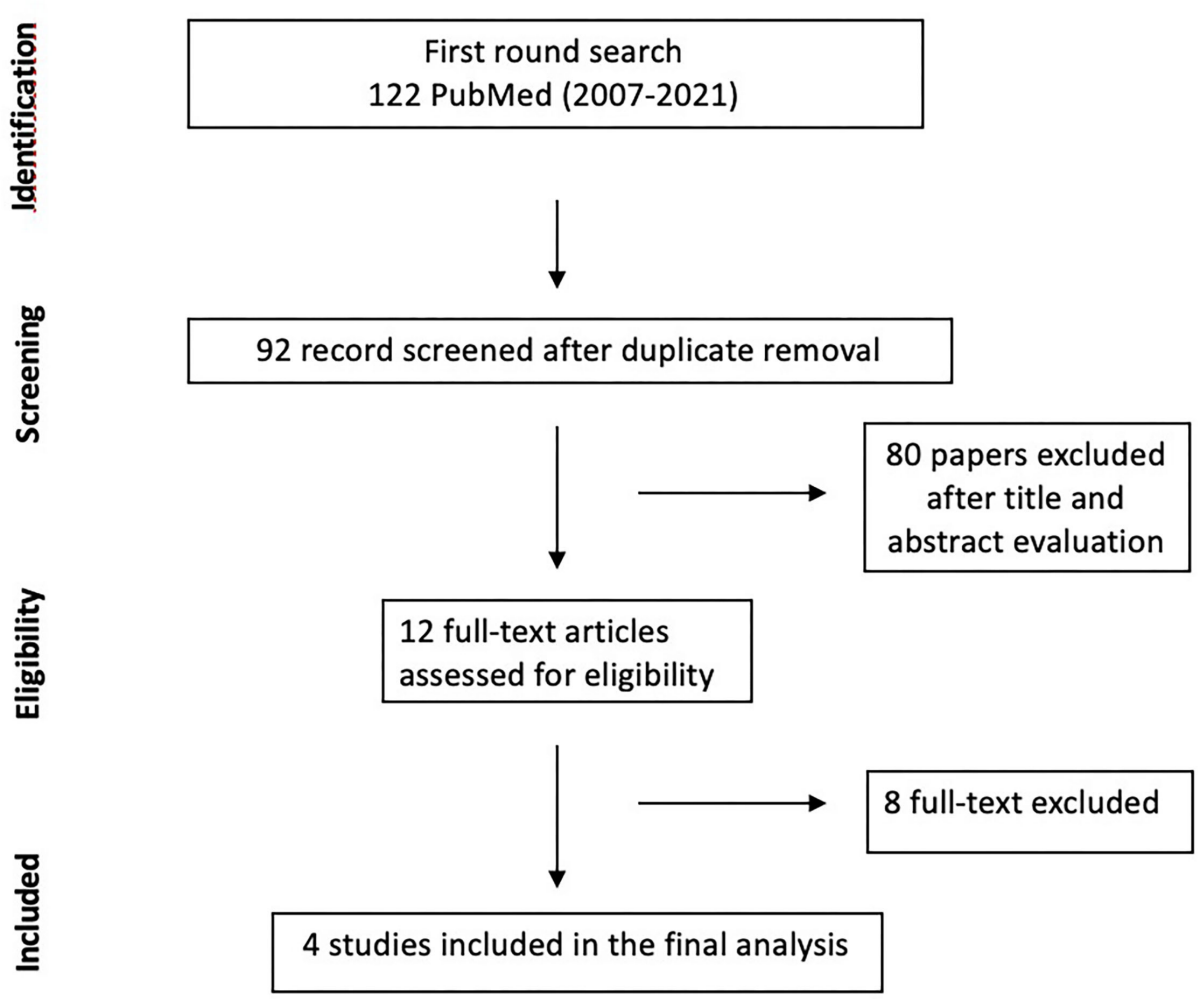
Flow chart of Search strategy divided by identification, screening, eligibility, included.

A total of 130 Abstract were screened in the first review round, after duplicates removal, and 12 papers were considered for full-text analysis. After excluding with reason eight manuscripts (Table 1), four paper were included in the present meta-analysis (10, 11, 19, 20) (Figure 1, Table 2). From the included studies, 525 patients were included in the Carmustine wafer group (Experimental Group), and 753 in the standard protocol group (Control Group).

**Table 1:** Studies excluded from the analysis.

**First author, year of publication, journal**	**Reason for exclusion**
Westphal et al., 2003, Neuro Oncol (9)	Recurrent glioblastoma multiforme
Attenello et al., 2008, Ann Surg Oncol (25)	Glioma grade III and IV were included
Salmaggi et al., 2013, Journal of Neurosurgery (12)	Not including standard treatment (surgery + chemo-/radio-therapy) group for comparison
Jungk et al., 2016, BMC Cancer (13)	Included recurrent glioblastoma cases only, Not including Carmustine Wafer treatment group for comparison
Della Puppa et al., 2017, J Neurooncol (14)	Not including NON-Carmustine Wafer treatment group for comparison
Champeaux et al., 2019, Journal of Neuro-Oncology (22)	Not including standard treatment (surgery + chemo-/radio-therapy) group for comparison
Ius et al., 2020, Cancer (2)	Not including Carmustine Wafer treatment group for comparison
Iuchi et al., 2022, *Neurooncol Adv* (21)	Not including NON-Carmustine Wafer treatment group for comparison

**Table 2:** Characteristics of included studies.

**Authors, year, journal**	**Type of study**	**Patients with/without CWs**	**Adjuvant therapy**	**Grade of Glioma**	**Molecular markers**	**EOR**	**OS results**	**PFS results**	**Side effects**
De Bonis et al.,2012, *Acta Neurochir (Wien)* (19)	Randomized controlled trial	10/67	Adjuvant therapy with TMZ	IV	NA	Non volumetric study	Adding CWs to standard treatment did not significantly improve the outcome Multivariate analysis showed the only was resection extent (*p* = 0.048)	NA	The toxicity after CW use was significantly higher, both for patients with newly diagnosed and patients with recurrent glioblastoma
Pallud et al., 2015, *Neuro Oncol* (10)	Randomized controlled trial	354/433	Chemoradiation standard protocol	IV	NA	Surgical resection at progression whether alone or combined with CW implantation was independently associated with longer overall survival in the whole series (*p* = 0.0001)	The median overall survival was 20.4 months and 18.0 months in the CWs group and non CWs group respectively	The median PFS was 12.0 months and 10.0 months in the CWs group and non CWs group respectively	The higher postoperative infection rate in the implantation group did not affect survival
Roux et al., 2017, *J Neurooncol* (11)	Randomized controlled trial	123/217	Standard combined chemoradiotherapy	IV	NA	Volumetric estimation In CWs group and non-CWs group the Subtotal (90% and >) and total (100%) removal were achieved in 55.6 and 55.1% of cases, respectively (*p* = 0.887)	CWs implantation was were independently associated with longer OS (*p* = 0.029)	CWs implantation was were independently associated with longer PFS (*p* = 0.045)	CWs did not significantly increase postoperative complications, including postoperative infections (*p* = 0.269, and *p* = 0.446, respectively)
Akiyama et al., 2018, *World Neurosurg* (20)	Randomized controlled trial	25/29	Standard combined chemoradiotherapy	IV	Evaluation of the IDH-1/2 mutation, which has been reported as a predictive factor, was performed in only a small percentage of patients	Volumetric estimation The median EOR was 93% in CWs group vs. 96% in non CWs gruop (*p* = 0.129)	The median OS in the CWs group and non CWs group was 24.2 months and 15.30 respectively (*p* = 0.027)	The median PFS in the CWs group and non CWs group was 16.8 months and 7.30 months, respectively (*p* = 0.009)	The incidence of adverse events were similar between the treatment groups, except for infection that was more common in the CWs patients (3.5% vs. 0%)

*CWs, Carmustine Wafers, EOR, extent of resection, NA, not applicable, PFS, Progression-free survival, OS, overall survival*.

### Overall Survival

**Figure 2:**
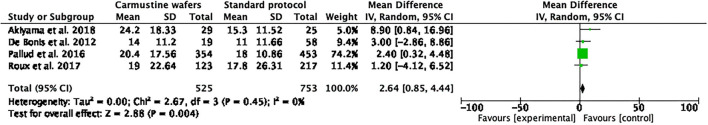
Foster plot—overall survival (*OS*). The *OS* of all included patients demonstrating that there was a significant advantage in using Carmustine Wafers (CWs) in newly diagnosed glioblastoma (GBM) in terms of *OS* and low heterogeneity in all included studies.

Quantitative data on *OS* were reported for all of the included patients (10, 11, 19, 20). The analysis of the meta-data demonstrated that there was a significant advantage in using CWs in newly diagnosed GBM in terms of *OS*, and a very low heterogeneity among the included studies (mean difference 1,492.64 (95% *CI*: 0.85, 4.44); *p* = 0.004; *I*^2^ = 0%; Figure 2).

### Progression Free Survival

**Figure 3:**
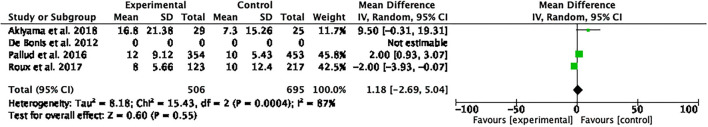
Foster plot—progression-free survival (*PFS*). The analysis of meta-data demonstrated that there were no significant differences between the two treatment groups in terms of *PFS*, even though a high heterogeneity should be considered.

The quantitative data on PFS were reported in three (10, 19, 20) out of the four included studies, which was based on a total of 171 patients in the Experimental group and 300 in the Control Group. The analysis of meta-data demonstrated that there were no significant differences between the two treatment groups in terms of PFS, even though a high heterogeneity must be considered mean difference [1.18 (95% *CI* −2.69, 5.04); *p* = 0.55; *I*^2^ = 87%; Figure 3].

### Complications' Rate

The complication rate was reported in three (11, 15, 16) out of the four included studies. This rate was 25.73% in the CWs group and 18.33% in the non-CWs group. The analysis of complications showed a relatively higher rate in carmustine-implanted patients, although this difference was not significant (*p* = 0.53).

## Discussion

Despite extensive resection, HGG remains virtually an uncurable disease because of the tendency of diffuse infiltrative growth beyond the radiological tumor borders (2, 4, 6–8). The current standard of care is based on combined maximal safe-resection and concomitant radiation and alkylating chemotherapy (1, 26).

After decades of research in therapeutic and molecular refinements, the traditional multimodal approach still leads to a mean survival rate of 14–16 months, with a 2-year survival rate of 26.5%; and <10% of patients alive 5 years after diagnosis (27).

In 2003, the intraoperative treatment with CWs implantation in newly HGG was introduce as a therapeutic bridge during the period between tumoral surgical resection and chemoradiotherapy onset, with the aim of interfering with the potential tumor growth at resection margins (5, 9–14). Different studies demonstrated a promising result in terms of PFS without a marked increase in toxicities as compared with the Stupp regimen. However, the gain in median survival using this schedule was less clear (10–12, 14, 19).

After an initial promising success, CWs implantation in HGGs have been gradually abandoned in day-to-day clinical practice since 2017 for several reasons. A specific position that is totally against the use of CWs is not reported in current literature. In a recent intersociety SNO-EANO (Society for Neuro-Oncology- European Society of Neuro-Oncology) consensus review, Wen et al. (17) summarized the current status of the treatment of newly diagnosed glioblastoma. With regards to the CWs, the authors stated that this treatment option provides a modest survival advantage of approximately 2 months It tends to be considered only in sporadic cases, mainly because issues related to risks involving safety and tolerability, in addition to the precluded enrollment of in other clinical trials in subsequent trials for the possible confounding effects generated by CWs. These points do not prevent or forbit the use of this treatment, however, provides indirect discouragement.

Recent long-term follow-up investigations, however, have shown survival benefits in newly HGG treated with CWs implantation, shedding light, for a second time, on the effectiveness of treatment with CWs.

### Overall Survival and Progression-Free Survival

The presents systematic review and meta-analysis, based on the comparative studies on CW effectiveness, demonstrates a significant advantage in using CWs in newly diagnosed GBM in terms of *OS*, but not in terms of PFS.

Conversely the propensity-matched French multicenter cohort study stated opposite conclusions, reporting that CWs implantation was independently associated with longer *PFS* in patients with subtotal/total surgical resection in the entire series (*p* = 0.005) and after propensity matching (*p* = 0.008) (10). In addition, the authors evidenced that there was no benefit for CWs implantation unless maximal resection was achieved. The role of extent of resection (*EOR*) in improving *OS* in patients with GBM has widely been demonstrated, with more extensive resections providing added survival benefits (1, 2, 5, 10, 11). To optimize the *EOR*, especially in deep fields or in conditions of non-orthogonal working corridors, the effectiveness of 5-ALA-guided surgery has been proven in volumetric investigations (28). In a level 2B evidence investigation, 5-ALA-assisted surgery intraoperative fluorescence was shown to be more effective than conventional surgery in increasing EOR and prolonging, thus *OS* in GBM patients (29). Della Puppa et al., further demonstrated that on GBM patients, 5-ALA technology and CW implantation provided a synergic action on patient outcomes without increasing adverse events occurrence, highlighting the importance of adequate patient selection.

Subsequently, Roux et al. concluded that wafer implantation in combination with maximal resection, followed by standard combined chemoradiotherapy is safe, efficient, and well-tolerated in newly diagnosed supratentorial glioblastomas in adults. Moreover, unlike the French study, in which the volume analysis was categorical, Roux includes a quantitative analysis emphasizing the maximum efficacy of CWs for lesions with *EOR* > 90% [adjusted hazard ratio (*HR*), 0.52 (95% *CI* 0.38–0.70), *p* < 0.001] (11).

Despite the lack of comparative analysis, Ius et al. found a longer survival in a CW subgroup of patients with *EOR* ≤ 100%. Enhanced survival benefits among CWs patients were observed in those patients with a higher percentage of methylated MGMT promoter, lower age, and total resection, thus highlighting several prognostic factors that could be evaluated in the selection process of patients with potentially better chances of postoperative success (5). On the bases of these results, an appropriate pre-operative patient screening based on the development of cell-free plasma DNA techniques to detect the methylation status of the MGMT promoter could prove to be important to preoperatively select young patients with small lesions that could potentially benefit from CWs implantation (30, 31).

Iuchi et al. (21) recently detected that CWs implantation in younger patients with an *EOR* >95% significantly prolongs the *OS* (median = S 27.4 months, 2-year *OS* = 46%). This latter investigation supports the criticism related to the effectiveness of CWs underlined by Champeux et al. (22) in a 9-year nationwide retrospective study in which the author found that the increase in *OS* after CW implantation was affected by age, gender, extent of surgery, and postoperative complications.

It is important to assess all potential treatment benefits of this treatment in selected HGG patients, even if literature in this field centers on the limits of this option when considered in HGG patients in general. Perhaps the comprehensive efficacy of this treatment should be reassessed in subpopulations of newly HGG patients.

### Side Effects and Surgical Considerations

The high number of adverse events reported in the literature has certainly limited the use of CWs in newly HGG patients (5, 10–17). The various complications, however, vary considerably among different investigations. These reported complications include malignant cerebral edema, resection cavity cyst formation, cerebrospinal fluid leak, wound healing abnormalities, and increased perioperative seizure activity. In this study, the overall complication rate was 25.73% in the CWs group (44 of 171 patients), while 18.33% in the standard treatment (55 of 300 patients; *p* = 0.53).

In a large meta-analysis, Bregy et al. (15) reviewed 19 studies based on a total of 795 patients, and reported a complication rate of 42%. Contrary results, however, were reported in 2008 by Attenello et al. (25) that retrospectively analyzed a cohort of more than 1,000 patients (including 288 patients implanted with CWs) and found that the morbidity rate between the CWs and non-CWs groups was similar, despite patients being slightly older in the CWs group. The efficiency and safety of CWs in newly diagnosed supratentorial glioblastomas in adults were also demonstrated by Roux et al. (11). Interestingly, De Bonis et al. (19) listed a statistically significant higher risk of side-related toxicity in patients treated for tumor recurrence, emphasizing the importance of patient selection.

Major studies agree on the importance of an adequate surgical technique to reduce the risk of common side effects (10, 11).

The most commonly observed postoperative complications are due to infection and development of hydrocephalus. Hydrocephalus tends to be caused by migration of wafers or inflammatory response to CWs diffusion through the defect. Implantation of CWs is not recommended in patients that involve the surgical opening of the ventricular system, considering that acute occlusive hydrocephalus can be brought on by the dislocation of the wafers into the ventricular system and ventriculitis in association with transient hydrocephalus (32, 33).

### Limitations

The interpretation of this present investigation should be considered in light of several limitations. The principle drawback concerns the information on the type of treatment carried out at tumor recurrence. It was difficult to assess whether the best *OS* in CWs patients was determined solely by CWs or by alternative treatments at the time of progression. It would be thus useful in future studies to evaluate the opportunity of exploring the survival benefits of salvage treatments, considering these covariates both time-dependent and fixed. Longer PFS, however, resulted in late tumor recurrence and consequently in better *OS* (34).

Another important issue contributing to reluctance to use CWs involves the lack of reliable survival data for patients treated with CWs, which might lead to confusion during the statistical analysis of the survival data of patients in a given trial. Moreover, it is well-known that to strengthen the survival benefit, salvage treatment information should ideally be included in the analysis at the time of tumor progression. The lack of standardized protocols for treatments at tumor progression represents thus an additional drawback. Overall, in future studies it would be useful to include the type of treatment at recurrence, considering this covariate both time-dependent and fixed to further render the survival data as a combination of all selected treatments used during the disease history.

With regards to the four investigations selected for the meta-analysis, raw data regarding the EOR in different subgroups were unfortunately not retrievable and thus was a limit of this study.

In addition, the majority of studies enrolled patients with Grade III and IV Gliomas, without stratifying the survival results according to the molecular profile or histological class, generating potentially confusing results.

In closing, in light to the novel 2021 WHO classification (35), it is important to integrate the volumetric data and the CDKN2A/2B, ATRX, TERT, EGFR, and TP53 status in future survival analysis to detect different categories of responders to a specific treatment protocol.

## Conclusions

The results of this meta-analysis seem to suggest that CWs implantation plays a significant role in improving survival when used in patients with newly diagnosed HGG. To minimize the risk of side effects, however, a careful patient selection should be considered, i.e., younger patients with a high probability of radical resection for small lesions (5). The predictive molecular biomarkers for Carmustine efficacy need to be investigated in future studies to better identify those patients that could benefit from this treatment option. Considering the crucial role of tumor microenvironment (TME) on the GBM progression (6, 7), the transcriptomic profile of cells representing the TME of patients responsive and not responsive to CW implantation could provide new insights in an appropriate patient selection.

## Data Availability Statement

The raw data supporting the conclusions of this article will be made available by the authors, without undue reservation.

## Author Contributions

Data curation and writing—original draft: AM, AP, DC, IM, and ST. Methodology and writing–review and editing: LR and TI. Formal analysis: LR and ST. Supervision: TI, AR, and MM. Validation: TI, AR, ST, and MM. All authors have read and agreed to the published version of the manuscript.

## Funding

This work has been supported by Progetto Ministero 291 della Salute, Giovani Ricercatori 2016 GR-2016-02364678. Application of GLIADEL wafers (BCNU, Carmustine) followed by temozolomide and radiotherapy in patients with high-grade glioma: a precision medicine based on molecular landscape. CUP: J26C16000000005.

## Conflict of Interest

The authors declare that the research was conducted in the absence of any commercial or financial relationships that could be construed as a potential conflict of interest.

## Publisher's Note

All claims expressed in this article are solely those of the authors and do not necessarily represent those of their affiliated organizations, or those of the publisher, the editors and the reviewers. Any product that may be evaluated in this article, or claim that may be made by its manufacturer, is not guaranteed or endorsed by the publisher.

## Abbreviations

BCNU: Bis-ChloroethylNitrosoUrea
CWs: Carmustine Wafers
EOR: extent of resection
HGG: high grade glioma
HR: hazard ratio
GBM: glioblastoma
OS: overall survival
PFS: progression-free survival.
